# Rehabilitation of a Partial Nasal Defect with Facial Prosthesis: A Case Report

**DOI:** 10.5681/joddd.2014.046

**Published:** 2014-12-03

**Authors:** Ramin Negahdari, Alireza Pournasrollah, Sepideh Bohlouli, Alireza Sighari Deljavan

**Affiliations:** ^1^Dental and Periodontal Research Center, Tabriz University of Medical Sciences, Tabriz, Iran; ^2^Assistant Professor, Department of Prosthodontics, Faculty of Dentistry, Tabriz University of Medical Science, Tabriz, Iran; ^3^Post graduate Student, Department of Oral Medicine, Faculty of Dentistry, Tabriz University of Medical Science, Tabriz, Iran; ^4^General Dentist, Faculty of Dentistry, Tabriz University of Medical Science, Tabriz, Iran

**Keywords:** Basal cell carcinoma, maxillofacial prosthesis, midface defects, nasal prosthesis, silicone prosthesis

## Abstract

>Malignancies of the midface result in cosmetic deformities that make maxillofacial prosthesis as an integral part of the treatment plan. Facial defects can be devastating in their impact on physical structure and function of the affected individual, leading to potentional compromises in quality of life. Reconstruction of nasal defects is a challenge for the prosthodontist because of esthetic and retention problems associated with the facial prosthesis. This paper reports the rehabilitation of a partial nasal defect caused by basal cell carcinoma treatment using a nasal prosthesis made with silicone elastomers and mechanical and anatomical retentive aids. The patient had no problem with the prosthesis, except for a partial loss of extrinsic coloration in the two-year follow-up.

## Introduction

Maxillofacial defects refer to any tissue loss of the face caused by trauma, burns, tumoral lesions and malignant disease.^1^ Basal cell carcinoma is a malignant disease that arises in the basal cell layer of the epidermis. The disease is usually triggered by damage to the skin caused by sunrays. Basal cell carcinoma of the nasal area has a high cure rate of more than 95% but a delay in seeking treatment can allow the cancer to enlarge, causing possible disability. Treatment of basal cell carcinoma of the nasal vestibule varies depending on the size, depth, and location of the cancer. Treatment options are surgical removal, chemotherapy, and radiation.^2^ Loss of structural continuity in the face can compromise speech, eating, swallowing, esthetics, and social relationship.^3-6^ Esthetic reestablishment is the most important purpose in reconstruction of maxillofacial defects.^7^ Patients with cured midface malignancies but no reconstruction of surgical defects with facial prostheses is not considered successfully treated.^6^ The nose is the most prominent feature of the face. The importance of the nose in facial harmony has been well recognized. Patient acceptance for the facial prosthesis is a challenging issue, substantially due to unrealistic patient expectations. According to the clinical experiences, the nasal prosthesis have the highest level of acceptance but the orbital and other facial prosthesis have a limited acceptance.^8,9^ As for the critical role of retention in success of facial prostheses, full consideration must be given to it.^10,11^ Anatomic undercuts, secondary mechanical factors, skin adhesives, and the implants are reported to enhance retention.^12,13^ In this report, a definitive nasal prosthesis has been used for rehabilitation of a partial nasal defect, using anatomic retentive aids and skin adhesives.

## Case Report 

**Figure 1.**
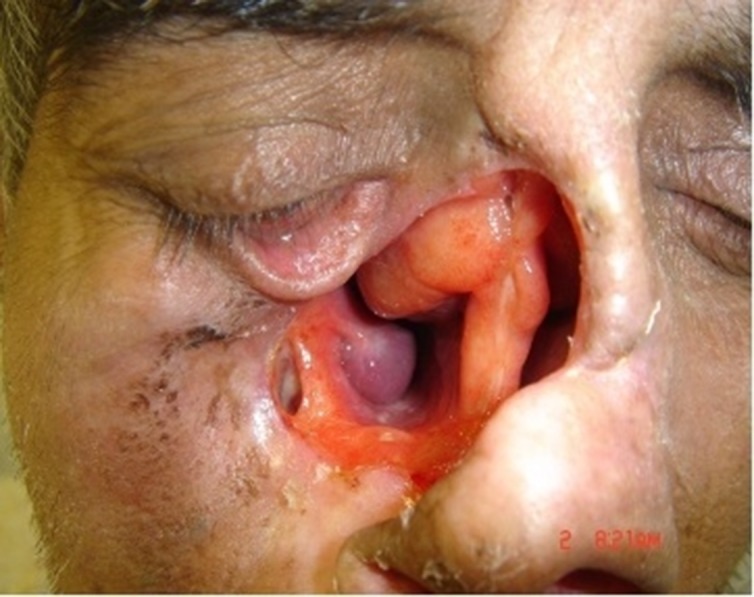
Pre-treatment facial view.

**Figure 2.**
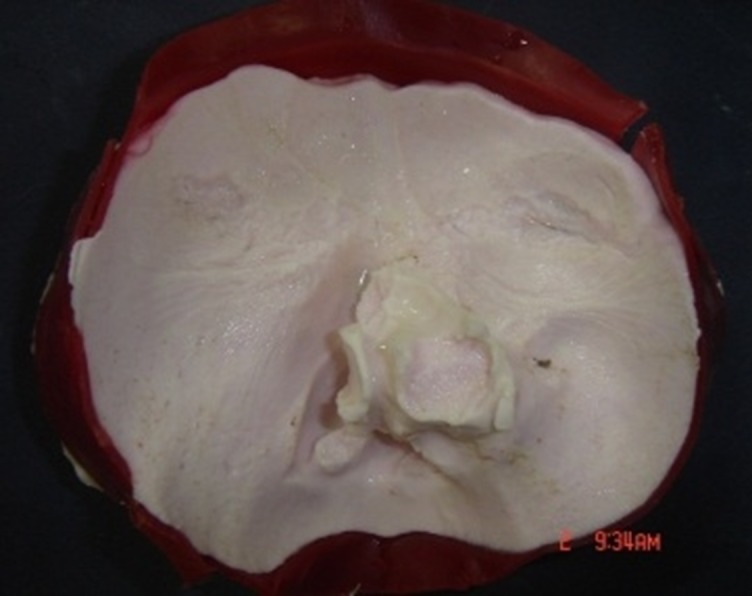
Impression of the defect area.

**Figure 3.**
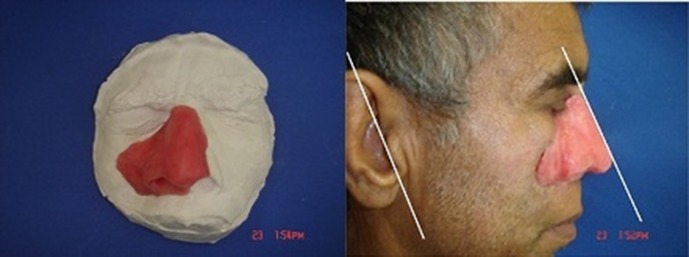
Wax pattern try-in of the prosthesis.

**Figure 4.**
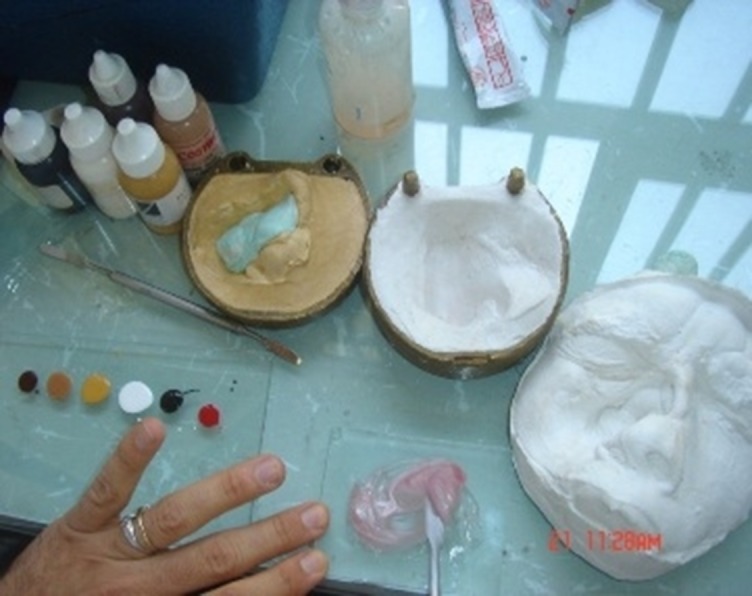
Base shade selection.

**Figure 5.**
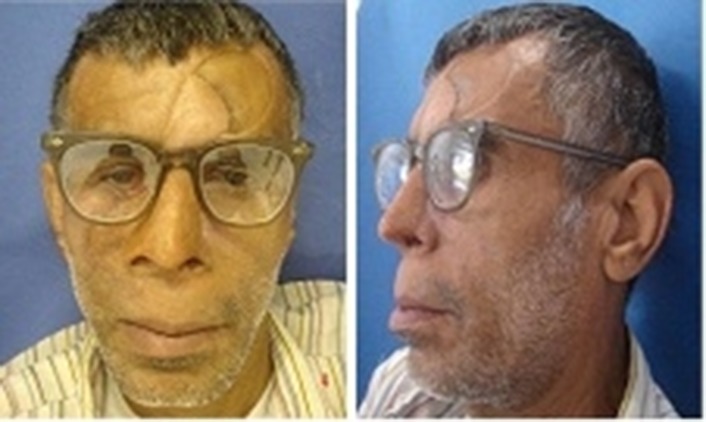
Frontal (left) and lateral (right) views of patient with final nasal prosthesis.

A fifty-four year-old man was referred to Tehran Cancer Institute, with a history of multiple recurrences of basal cell carcinoma (BCC) in the nasal septum and right nasal wall (Figure 1). After surgical resection, radiotherapy was initiated for the facial defect consisting of 50 Gy in 25 fractions. Six months after radiotherapy the boundary for the impression was outlined on the face and an impression was taken with an irreversible hydrocolloid impression material (Alginate; Tropicalgin, Zermack, Rovigo, Italy; Figure 2). The irreversible hydrocolloid was reinforced with gauze and dental plaster. The impression was poured in dental stone (Moldano; Bayer, Leverkusen, Germany). The wax pattern of the nose was sculpted on the plaster cast with dental base plate wax (Trubyte; Dentsply, York, USA). After the completion of the wax pattern, in order to improve the whole morphology of the nose on the face, we verified the contour, surface texture and the position of the wax pattern, during try-in procedure. The wax pattern sculpting procedure was done according to preoperative photographs of the patients in straight form. The position of the nostrils was verified with the inner canthus distance of the eyes. Nose profile matched the line between the ear’s top point and bottom of tragus (Figure 3).^7^ In order to improve the marginal adaptation of the wax pattern, it was relined with an elastomeric impression material (Speedex; Coltene AG, Switzerland). After verification of the shape, size, contour, fit, and surface texture of the corrected pattern on the face and ensuring that it is acceptable to both the patient and the practitioner, the mold was fabricated to reproduce the wax pattern in silicone. The molding procedure was carried out and the silicone elastomers (Cosmesil RTV) were colored intrinsically with Intrinsic Coloring Kit (Factor II Inc., Lakeside, AR, USA) on the face to match different shades of the patient’s skin (Figure 4). Approximately 100 gr of silicon materials with the selected base color was mixed to fill the mold. The target base color was selected according to the overall skin tone without characterization from the lightest area of the skin. Small amounts of red, blue, yellow, white, and green pigments was added conservatively to the mixed silicon and blended thoroughly to match the selected base color for the skin. Opacity and value of the silicon was increased with small amounts of kaolin. Trace amounts of red and yellow pigments were used to achieve closer match to the skin. Blue pigment was added to reduce the value. Layers of laminar glazes painted onto the mold for illustrating the histologic structure of the skin; then colored silicone was filled into the mold and light pressure was applied to the mold for removing excess material. The mold was then transferred to clamp and placed into a dry-heat oven at the manufacturer’s prescribed polymerization time and temperature. After the polymerization cycle was completed, the mold was allowed to cool in room temperature. Then the prosthesis was removed carefully from the mold, excess material was trimmed with scissors to make the prosthesis more esthetically acceptable. Appearance of the prosthesis was improved with extrinsic coloring and using eyeglasses. The prosthesis was delivered to the patient and home care instructions were given. Medical grade skin adhesive (Pros-Aide; FX Warehouse Inc, Philadelphia, USA) was used to enhance the retention of prosthesis. Periodic follow-ups were scheduled one month, three months, six months and one year after. The patient reported to be comfortable with the prosthesis (Figure 5). The patient gave consent to the publication of the treatment report including the full face figures.

## Discussion

After surgical removal of the tumors of the midfacial region, the patient is restricted from daily social activities. For patient with facial defects, one treatment choice is plastic and reconstructive surgery, but for larger defects with extensive anatomical loss, when surgical approach is not a feasible option, the prosthetic rehabilitation is considered the best choice.^6^This article described the steps for fabrication and construction of a large nasal prosthesis using the available materials. The treatment objectives for such patients are to reconstruct the lost tissues as soon as possible after surgery to maintain appearance, morale and self-confidence of the patient and improve social relations among the public and their families. One challenge in such cases is the retention of facial prosthesis and therefore the interdisciplinary collaboration of the prosthodontist and maxillofacial surgeon can assist in preserving the anatomic sites with critical role in retaining and supporting of the facial prosthesis. For instance, the preservation of the nose bridge can provide sufficient support for both a nasal prosthesis and eyeglasses frame.^14,15^ Retention methods include mechanical, anatomic, adhesive and implant-assisted. The decision on the type of the retention should be made based on the size and the position of the defect. In the reported case, there was a medium mid-facial defect with anatomic undercuts, and the application of the adhesive was sufficient for maintaining the prosthesis in place. As a further advantage, the adhesive application can seal the margins and prevent the air leakage and secretions from the nasal defect. The stability and the retention of the prosthesis was acceptable; however, eyeglasses were offered to the patient to mask the margins of prosthesis and further increase the retention.

The silicones are the most widely used material for maxillofacial prostheses because of advantages like light weight, softness, life-like appearance^7,16^, tranlucensy, possibility of intrinsic and extrinsic coloration, dimensional stability, flexibility, natural skin-like texture, and no allergic responses.^17^ For the present case, a silicone material with intrinsic coloring was used and in order to achieve a natural appearance, further extrinsic coloring was applied. The intrinsic coloration increases the color stability and translucency of the prosthesis.

Another more reliable alternative to restore facial defects is the use of extra-oral implants. Their success in improving the retention of facial prostheses has been well-documented; however, this modality requires the presence of adequate bone. Furthermore, in the case of patients with recurrent tumors, a longer observation and oncologic follow-up period is required before implant insertion.^14-17^

On the other hand, some of the problems with the use of adhesives for retention is poor bond strength with certain materials, unpredictable periods of retention in daily use, and causing the prosthetic material to degrade especially on its borders where the material is thinner. Another problem is movements of soft tissues around the midfacial defects during smile and other facial functions, which can compromise the adaptation of the margins.

In this case, a waxing process was used for constructing the prosthetic model. Laser scanning, CAD/CAM, and rapid prototyping technologies are the other methods which can simplify the model fabrication. Because the entire process can be automated, the CAD/CAM process decreases the number of manual steps.^16-18^Use of adhesive for the prosthesis is cost effective, non-invasive and without aggressive side effects, so it is easily accepted by the patients and their families. Patients must be instructed to remove the prosthesis once daily to clean underlying and peripheral tissues. They should be advised to remove the prosthesis at night before going to bed, in order to limit the risk of contact irritation of the skin.^14,18^

Such prostheses are acceptable to the patients because of their ease of use, low weight and good appearance.
